# Supplementary figures and images for: Socioeconomic inequalities, psychosocial stressors at work and physician-diagnosed depression: Time-to-event mediation analysis in the presence of time-varying confounders
Source: PLoS One. 2023 Oct 25;18(10):e0293388. doi: 10.1371/journal.pone.0293388 (PMC10599565; doi:10.1371/journal.pone.0293388)

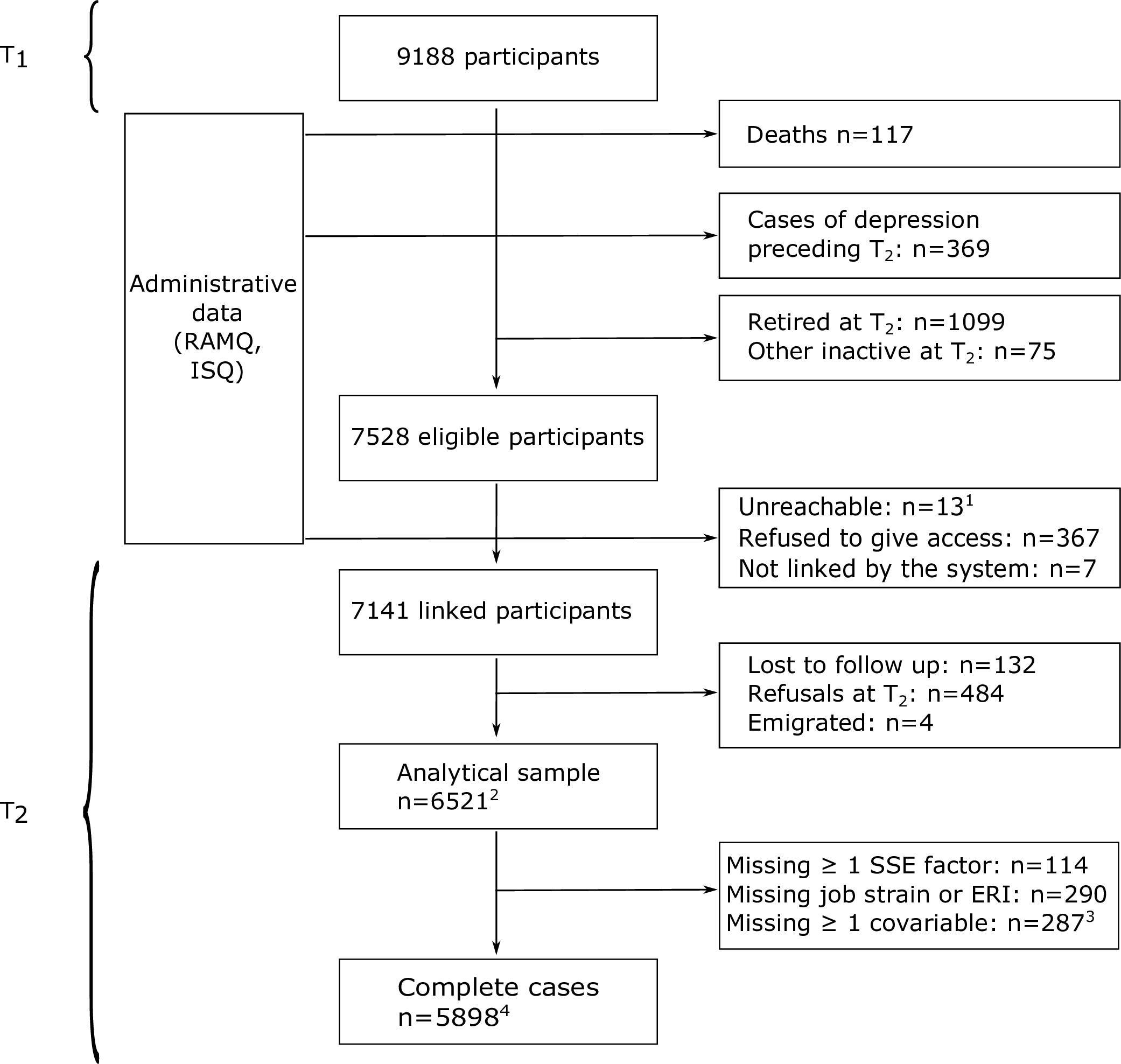

Supplement: S1 Fig — 1 Participants missing the necessary information to be re-contacted at follow-up. 2 6521/7528 = 86.6% of eligible at T2; 6521/9188 = 71.0% of baseline; men: 3217, women: 3304 (50.7%). 3 Not mutually exclusive, total = 623 participants. 4 5898/7528 = 78.3% of eligible at T2; 5898/9188 = 64.2% of baseline; men: 2963, women: 2935 (49.8%). (TIF) [file pone.0293388.s001.tif]

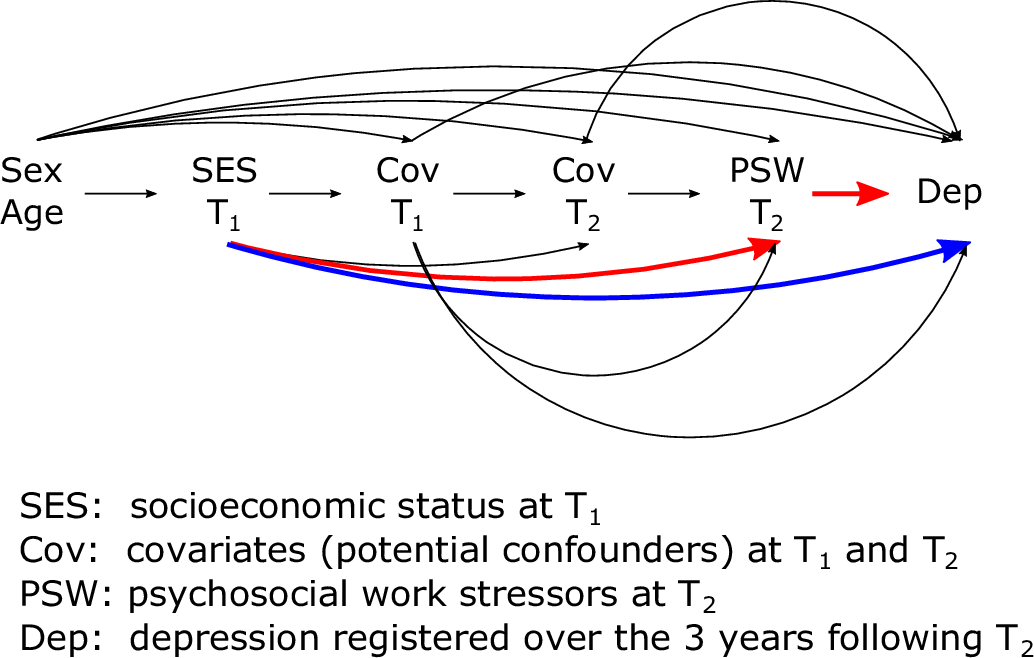

Supplement: S2 Fig — SES: Socioeconomic status at T1. Cov: Covariates (potential confounders) at T1 and T2. PSW: Psychosocial stressors at work at T2. Dep: Depression registered over the 3 years following T2. (TIF) [file pone.0293388.s002.tif]
