# Supplementary material for: Socioeconomic inequalities, psychosocial stressors at work and physician-diagnosed depression: Time-to-event mediation analysis in the presence of time-varying confounders
Source: PLoS One. 2023 Oct 25;18(10):e0293388. doi: 10.1371/journal.pone.0293388 (PMC10599565; doi:10.1371/journal.pone.0293388)
Supplement: S1 Table — a Three levels as in main analyses (1: < 40 000 CAD, 2: 40 000–70 000 CAD, 3: ≥ 70 000 CAD). b Three levels as in main analyses (1: Without college, 2: 2 years college, 3: Bachelor). c Three levels as in main analyses (1: Others, 2: professionals, 3: Managers). For all further analyses, component 1 was used as combined measure of socioeconomic status. (PDF) [file pone.0293388.s003.pdf]

S1 Table. Combined measure of socioeconomic status using principal component analysis (n = 5898 complete cases).

	**Component 1**	Component 2	Component 3
Household income[a]	**0.50**	0.83	-0.26
Education level[b]	**0.69**	-0.56	-0.46
Occupation type[c]	**0.52**	-0.05	0.85
Variation explained	**64.8%**	23.4%	11.9 %

[a] Three levels as in main analyses (1: < 40 000 CAD, 2: 40 000-70 000 CAD, 3: ≥ 70 000 CAD).

[b] Three levels as in main analyses (1: without college, 2: 2 years college, 3: bachelor).

[c] Three levels as in main analyses (1: others, 2: professionals, 3: managers).

For all further analyses, component 1 was used as combined measure of socioeconomic status.
