# Supplementary material for: Socioeconomic inequalities, psychosocial stressors at work and physician-diagnosed depression: Time-to-event mediation analysis in the presence of time-varying confounders
Source: PLoS One. 2023 Oct 25;18(10):e0293388. doi: 10.1371/journal.pone.0293388 (PMC10599565; doi:10.1371/journal.pone.0293388)
Supplement: S2 Table — (PDF) [file pone.0293388.s004.pdf]

S2 Table. ICD-9 codes for identifying cases of physician-diagnosed depression

ICD-9	n	Diagnostic
296.0	4	Bipolar disorder, single manic episode
296.1	7	Manic disorder recurrent episode
296.2	6	Major depressive disorder, single episode
296.3	0	Major depressive disorder, recurrent
296.4	1	Bipolar disorder, most recent episode (or current) manic
296.5	1	Bipolar disorder, most recent episode (or current) depressed
296.6	5	Bipolar disorder, most recent episode (or current) mixed
296.7	0	Bipolar disorder, most recent episode (or current) unspecified
296.8	0	Other and unspecified bipolar disorders
296.9	9	Unspecified episodic mood disorder
300.4	122	Dysthymic disorder
311	314	Depressive disorder, not elsewhere classified
