# Supplementary material for: Socioeconomic inequalities, psychosocial stressors at work and physician-diagnosed depression: Time-to-event mediation analysis in the presence of time-varying confounders
Source: PLoS One. 2023 Oct 25;18(10):e0293388. doi: 10.1371/journal.pone.0293388 (PMC10599565; doi:10.1371/journal.pone.0293388)
Supplement: S3 Table — S3a Table: Cohort characteristics at baseline (1991–1993) by educational level. S3b Table: Cohort characteristics at baseline (1991–1993) by household income. S3c Table: Cohort characteristics at baseline (1991–1993) by occupation type. (PDF) [file pone.0293388.s005.pdf]

S3a Table. Cohort characteristics at baseline (1991-1993) by educational level.

	Women				Men			
	**≤High school** 41.6% (n = 1375)	**College** 31.4% (n = 1036)	**≥University** 26.5% (n = 877)	**Total** (n=3304)	**≤High school** 12.2% (n = 393)	**College** 26.7% (n = 858)	**≥University** 60.6% (n = 1950)	**Total** (n=3217)
**Age (years)**	37.97 (5.68)	35.67 (7.18)	36.38 (6.12)	36.85 (6.40)	40.97 (5.86)	37.77 (7.15)	39.31 (6.91)	39.12 (6.92)
**Household income**								
< 40 000 CAD	521 (38.8)	350 (34.1)	151 (17.4)	1027 (31.6)	156 (39.8)	270 (31.6)	183 (9.4)	611 (19.1)
40 000 – 60 999 CAD	658 (49.0)	476 (46.3)	311 (35.9)	1452 (44.7)	194 (49.5)	443 (51.9)	861 (44.4)	1503 (47.0)
≥ 70 000 CAD	163 (12.1)	201 (19.6)	405 (46.7)	771 (23.7)	42 (10.7)	141 (16.5)	896 (46.2)	1087 (34.0)
**Occupation type**								
Manager	29 (2.1)	26 (2.5)	56 (6.4)	112 (3.4)	39 (9.9)	56 (6.5)	367 (18.9)	465 (14.5)
Professional	10 (0.7)	101 (9.8)	596 (68.1)	707 (21.5)	27 (6.9)	199 (23.2)	1379 (70.9)	1612 (50.2)
Others	1332 (97.2)	905 (87.7)	223 (25.5)	2474 (75.1)	327 (83.2)	602 (70.2)	200 (10.3)	1135 (35.3)
**Marital Status**								
Married/cohabiting	988 (72.3)	715 (69.5)	598 (68.6)	2314 (70.5)	317 (81.9)	659 (77.4)	1553 (80.2)	2544 (79.7)
**Children**								
Yes	925 (67.6)	561 (54.5)	467 (53.6)	1964 (59.8)	274 (70.1)	530 (62.2)	1335 (68.8)	2150 (67.2)
**Smoking**								
Never	531 (38.8)	484 (47.1)	450 (51.6)	1475 (44.9)	117 (30.1)	373 (43.7)	941 (48.6)	1435 (44.9)
Past smoker	428 (31.3)	303 (29.5)	256 (29.4)	990 (30.2)	163 (41.9)	290 (34.0)	677 (34.9)	1137 (35.6)
Current smoker	409 (29.9)	241 (23.4)	166 (19.0)	817 (24.9)	109 (28.0)	190 (22.3)	320 (16.5)	622 (19.5)
**Alcohol**								
≥ 6 doses/week	122 (8.9)	77 (7.4)	145 (16.6)	346 (10.5)	105 (26.8)	229 (26.8)	590 (30.4)	928 (29.0)
**Physical activity**								
≤ once/month	532 (38.8)	319 (30.9)	228 (26.0)	1087 (33.0)	135 (34.5)	237 (27.7)	419 (21.5)	795 (24.8)
≤ once/week	379 (27.7)	311 (30.1)	302 (34.5)	994 (30.2)	101 (25.8)	267 (31.2)	599 (30.8)	972 (30.3)
> once/week	459 (33.5)	403 (39.0)	346 (39.5)	1214 (36.8)	155 (39.6)	353 (41.2)	929 (47.7)	1443 (45.0)
**Job Strain**								
Yes	307 (22.7)	273 (26.5)	197 (22.6)	780 (23.8)	63 (16.2)	183 (21.4)	351 (18.1)	598 (18.7)

S3b Table. Cohort characteristics at baseline (1991-1993) by household income.

	Women				Men			
	**< $40 000**	**$40 000 – $69999**	**>$70 000**	**Total**	**< $40 000**	**$40 000 – $69999**	**≥ $70 000**	**Total**
	31.1% (n = 1027)	43.9% (n = 1452)	23.3% (n = 771)	(n=3304)	18.1% (n = 611)	47.2% (n = 1503)	34.8% (n = 1087)	(n=3217)
**Age (years)**	36.60 (7.23)	36.37 (6.09)	38.01 (5.49)	36.85 (6.40)	35.48 (7.56)	39.31 (6.57)	40.90 (6.17)	39.12 (6.92)
**Occupation type**								
Manager	15 (1.5)	34 (2.3)	63 (8.2)	112 (3.4)	8 (1.3)	147 (9.8)	308 (28.5)	465 (14.5)
Professional	79 (7.7)	258 (17.8)	362 (47.2)	707 (21.5)	111 (18.2)	825 (54.9)	668 (61.7)	1612 (50.2)
Others	928 (90.8)	1158 (79.9)	342 (44.6)	2474 (75.1)	492 (80.5)	531 (35.3)	106 (9.8)	1135 (35.3)
**Marital Status**								
Married/cohabiting	295 (29.0)	1231 (85.1)	750 (97.5)	2314 (70.5)	289 (47.9)	1221 (81.7)	1025 (95.0)	2544 (79.7)
**Children**								
Yes	453 (44.4)	935 (64.8)	551 (71.5)	1964 (59.8)	243 (40.0)	1039 (69.4)	860 (79.6)	2150 (67.2)
**Smoking**								
Never	446 (43.7)	645 (44.7)	363 (47.5)	1475 (44.9)	299 (49.6)	661 (44.3)	464 (42.8)	1435 (44.9)
Past smoker	278 (27.2)	456 (31.6)	241 (31.5)	990 (30.2)	154 (25.5)	560 (37.5)	419 (38.7)	1137 (35.6)
Current smoker	297 (29.1)	343 (23.8)	160 (20.9)	817 (24.9)	150 (24.9)	271 (18.2)	200 (18.5)	622 (19.5)
**Alcohol**								
≥ 6 doses/week	81 (7.9)	140 (9.7)	120 (15.6)	346 (10.5)	158 (25.9)	393 (26.2)	374 (34.6)	928 (29.0)
**Physical activity**								
≤ once/month	336 (32.8)	497 (34.3)	229 (29.8)	1087 (33.0)	172 (28.3)	381 (25.4)	239 (22.0)	795 (24.8)
≤ once/week	289 (28.2)	447 (30.9)	242 (31.5)	994 (30.2)	178 (29.3)	455 (30.3)	335 (30.8)	972 (30.3)
> once/week	399 (39.0)	504 (34.8)	298 (38.8)	1214 (36.8)	258 (42.4)	664 (44.3)	512 (47.1)	1443 (45.0)
**Job strain**								
Yes	254 (25.0)	345 (24.0)	169 (22.1)	780 (23.8)	123 (20.2)	273 (18.2)	195 (18.0)	598 (18.7)

S3c Table. Cohort characteristics at baseline (1991-1993) by occupation type.

	Women				Men			
	**Others** 76.7% (n = 2474)	**Professional** 20.3% (n = 707)	**Manager** 3.5% (n = 112)	**Total** (n=3304)	**Others** 35.3% (n = 1135)	**Professional** 50.1% (n = 1612)	**Manager** 14.5% (n = 465)	**Total** (n=3217)
**Age (years)**	36.85 (6.51)	36.31 (5.90)	40.01 (5.97)	36.85 (6.40)	37.50 (6.95)	38.91 (6.77)	43.68 (5.04)	39.12 (6.92)
**Marital Status**								
Married/cohabiting	1740 (70.7)	488 (69.5)	79 (71.2)	2314 (70.5)	833 (74.2)	1300 (81.1)	406 (88.3)	2544 (79.7)
**Children**								
Yes	1502 (61.0)	379 (54.0)	76 (67.9)	1964 (59.8)	634 (56.1)	1100 (68.7)	411 (88.6)	2150 (67.2)
**Smoking**								
Never	1056 (43.0)	367 (52.1)	48 (43.6)	1475 (44.9)	480 (42.6)	787 (49.2)	167 (36.1)	1435 (44.9)
Past smoker	737 (30.0)	217 (30.8)	32 (29.1)	990 (30.2)	383 (34.0)	543 (33.9)	208 (45.0)	1137 (35.6)
Current smoker	665 (27.1)	120 (17.0)	30 (27.3)	817 (24.9)	263 (23.4)	271 (16.9)	87 (18.8)	622 (19.5)
**Alcohol**								
≥ 6 doses/week	211 (8.6)	112 (15.9)	23 (20.7)	346 (10.5)	292 (25.8)	473 (29.4)	163 (35.4)	928 (29.0)
**Physical activity**								
≤ once/month	857 (34.8)	186 (26.3)	41 (36.9)	1087 (33.0)	342 (30.2)	339 (21.1)	114 (24.5)	795 (24.8)
≤ once/week	728 (29.5)	229 (32.4)	32 (28.8)	994 (30.2)	326 (28.8)	514 (31.9)	129 (27.7)	972 (30.3)
> once/week	881 (35.7)	292 (41.3)	38 (34.2)	1214 (36.8)	463 (40.9)	756 (47.0)	222 (47.7)	1443 (45.0)
**Job Strain**								
Yes	585 (23.9)	166 (23.5)	29 (26.1)	780 (23.8)	226 (20.1)	304 (19.0)	67 (14.4)	598 (18.7)
