# Supplementary material for: Socioeconomic inequalities, psychosocial stressors at work and physician-diagnosed depression: Time-to-event mediation analysis in the presence of time-varying confounders
Source: PLoS One. 2023 Oct 25;18(10):e0293388. doi: 10.1371/journal.pone.0293388 (PMC10599565; doi:10.1371/journal.pone.0293388)
Supplement: S4 Table — ndep: Number of cases of depression. PY: Person-years. All other values are HR, adjusted for age and for sex (last column). Bold: 95% CI that do not include 1. TSD: 1000 CAD$. Income is before tax household income per year. (PDF) [file pone.0293388.s006.pdf]

S4 Table. Socioeconomic gradient of depression in the three years after T2 (n = 5898 complete cases).

SES	Men, n=2963			Women, n=2935			Both, n=5898		
	n (n_{dep})	PY	HR (CI 95%)	n (n_{dep})	PY	HR (CI 95%)	n (n_{dep})	PY	HR (CI 95%)
**Education**									
Ref: university	1815 (85)	5314	1	807 (78)	2295	1	2622 (163)	7608	1
2 years college	805 (34)	2353	0.987 (0.648-1.416)	944 (87)	2698	0.931 (0.691-1.255)	1749 (121)	5051	0.963 (0.749-1.215)
no college	343 (25)	993	**1.676** (1.006-2.556)	1184 (113)	3367	0.990 (0.756-1.352)	1527 (138)	4360	1.223 (0.959-1.558)
**Income**									
Ref: ≥70 TSD	1017 (43)	2986	1	720 (61)	2062	1	1737 (104)	5047	1
40-70 TSD	1397 (67)	4076	1.172 (0.807-1.762)	1323 (112)	3791	0.983 (0.734-1.364)	2720 (179)	7867	1.037 (0.797-1.334)
< 40 TSD	549 (34)	1599	1.620 (0.978-2.731)	892 (105)	2507	1.311 (0.986-1.834)	1441 (139)	4106	**1.387** (1.076-1.834)
**Occupation**									
Ref: managers	435 (14)	1274	1	98 (8)	281	1	533 (22)	1555	1
professional	1504 (70)	4408	1.261 (0.730-2.643)	661 (1875)	1875	1.171 (0.613-3.161)	2165 (134)	6283	1.441 (0.931-2.498)
others	1024 (60)	2978	1.719 (0.966-3.629)	2176 (206)	6203	1.140 (0.616-2.961)	3200 (266)	9181	**1.592** (1.048-2.746)
**Comb. SES**									
Ref: high	922 (35)	2718	1	344 (26)	992	1	1266 (61)	3710	1
medium	1529 (79)	4444	1.421 (0.940-2.216)	1141 (107)	3246	1.264 (0.875-2.091)	2670 (186)	7690	1.317 (0.986-1.830)
low	512 (30)	1498	**1.697** (1.060-2.837)	1450 (145)	4121	1.323 (0.921-2.119)	1962 (175)	5620	**1.428** (1.033-2.051)

n_{dep}: number of cases of depression. PY: person-years. All other values are HR, adjusted for age and for sex (last column). Bold: 95% CI that do not include 1. TSD: 1000 CAD$. Income is before tax household income per year.
