# Supplementary material for: Socioeconomic inequalities, psychosocial stressors at work and physician-diagnosed depression: Time-to-event mediation analysis in the presence of time-varying confounders
Source: PLoS One. 2023 Oct 25;18(10):e0293388. doi: 10.1371/journal.pone.0293388 (PMC10599565; doi:10.1371/journal.pone.0293388)
Supplement: S5 Table — ndep: Number of cases of depression. PY: Person-years. All other values are HR. Model 1: Adjusted for age and for sex. Model 2: Adjusted for age, sex (last column), SES indicators at T1, family indicators (marital status, presence of children in the household) and lifestyle habits (smoking, alcohol consumption and leisure time physical activity) at T1 and T2. Bold: 95% CI that do not include 1. (PDF) [file pone.0293388.s007.pdf]

S5 Table. Effect estimates of psychosocial stressors at work on depression in the three years after T2 (n = 5898 complete cases).

Stressor	Men, n=2963				Women, n=2935				Both, n=5898			
	n (n_{dep})	PY	HR Model 1	HR Model 2	n (n_{dep})	PY	HR Model 1	HR Model 2	n (n_{dep})	PY	HR Model 1	HR Model 2
**Job strain**												
Ref: no strain	2517 (110)	7372	1	1	2314 (208)	6612	1	1	4831 (318)	13984	1	1
High strain	446 (34)	1289	**1.796** (1.187-2.566)	**1.758** (1.193-2.51)	621 (70)	1747	1.266 (0.953-1.650)	1.277 (0.964-1.649)	1067 (104)	3036	**1.435** (1.145-1.791)	**1.418** (1.102-1.766)
**ERI**												
Ref: no imbalance	2220 (94)	6505	1	1	2223 (184)	6385	1	1	4443 (278)	12890	1	1
Imbalance	743 (50)	2155	**1.613** (1.110-2.259)	**1.606** (1.104-2.272)	712 (94)	1975	**1.658** (1.277-2.112)	**1.664** (1.265-2.098)	1455 (144)	4130	**1.639** (1.341- 1.975)	**1.679** (1.378-2.021)

n_{dep}: number of cases of depression. PY: person-years. All other values are HR. Model 1: adjusted for age and for sex. Model 2: adjusted for age, sex (last column), SES indicators at T_1, family indicators (marital status, presence of children in the household) and lifestyle habits (smoking, alcohol consumption and leisure time physical activity) at T_1 and T_2. Bold: 95% CI that do not include 1.
