# Supplementary material for: Socioeconomic inequalities, psychosocial stressors at work and physician-diagnosed depression: Time-to-event mediation analysis in the presence of time-varying confounders
Source: PLoS One. 2023 Oct 25;18(10):e0293388. doi: 10.1371/journal.pone.0293388 (PMC10599565; doi:10.1371/journal.pone.0293388)
Supplement: S6 Table — All values are HR, adjusted for age. Bold: 95% CI that do not include 1. TSD: CAD 1000. Income is before-tax household income per year. (PDF) [file pone.0293388.s008.pdf]

S6 Table. Association between SES and psychosocial stressors at work in men (n = 2963 complete cases).

SES	Job strain T_2	Job strain T_2 adjusted for job strain in T_1	ERI T_2	ERI T_2 adjusted for job strain in T_1
**Education**				
Ref: university	1	1	1	
2 years college	0.992 (0.818-1.252)	0.956 (0.765-1.209)	**0.788** (0.654-0.909)	**0.768** (0.646-0.907)
no college	1.105 (0.791-1.500)	1.144 (0.878-1.574)	**0.599** (0.424 0.808)	**0.606** (0.434-0.740)
**Income**				
Ref: ≥70 TSD	1	1	1	
40-70 TSD	1.064 (0.796-1.328)	1.076 (0.874-1.345)	0.849 (0.715-1.020)	**0.852** (0.703-0.979)
< 40 TSD	**1.643** (1.246-2.208)	**1.658** (1.248-2.276)	0.959 (0.781-1.204)	0.954 (0.736-1.172)
**Occupation**				
Ref: managers	1	1	1	1
professionals	**1.568** (1.168-2.394)	**1.483** (1.030-2.240)	**0.745** (0.588-0.977)	**0.712** (0.577-0.889)
others	**1.830** (1.323-2.901)	**1.740** (1.143-2.697)	**0.748** (0.578-0.936)	**0.714** (0.552-0.911)

All values are HR, adjusted for age. Bold: 95% CI that do not include 1. TSD: CAD 1000. Income is before-tax household income per year.
