# Supplementary material for: Socioeconomic inequalities, psychosocial stressors at work and physician-diagnosed depression: Time-to-event mediation analysis in the presence of time-varying confounders
Source: PLoS One. 2023 Oct 25;18(10):e0293388. doi: 10.1371/journal.pone.0293388 (PMC10599565; doi:10.1371/journal.pone.0293388)
Supplement: S7 Table — All values are HR, adjusted for age. Bold: 95% CI that do not include 1. TSD: CAD 1000. Income is before-tax household income per year. (PDF) [file pone.0293388.s009.pdf]

S7 Table. Association between SES and psychosocial stressors at work in women (n = 2935 complete cases).

**SES**	**Job strain T_2**	**Job strain T_2** adjusted for job strain in T_1	**ERI T_2**	**ERI T_2** adjusted for job strain in T_1
**Education**				
Ref: university	1	1	1	
2 years college	**1.258** (1.032-1.594)	1.209 (0.957-1.493)	0.926 (0.749-1.140)	0.899 (0.700-1.076)
no college	1.123 (0.920-1.410)	1.119 (0.948-1.343)	**0.640** (0.527-0.757)	**0.632** (0.507-0.787)
**Income**				
Ref: ≥70 TSD	1	1	1	
40-70 TSD	**1.322** (1.101-1.625)	**1.330** (1.097-1.704)	0.796 (0.656-1.013)	0.790 (0.624-1.009)
< 40 TSD	**1.430** (1.165-1.864)	**1.402** (1.114-1.749)	1.004 (0.821-1.279)	0.984 (0.796-1.309)
**Occupation**				
Ref: managers	1	1	1	1
professionals	1.374 (0.641-2.680)	1.538 (0.824-2.781)	0.672 (0.455-1.070)	0.705 (0.431-1.329)
others	1.680 (0.889-3.417)	1.878 (0.975-3.775)	**0.628** (0.423-0.971)	0.653 (0.410-1.352)

All values are HR, adjusted for age. Bold: 95% CI that do not include 1. TSD: CAD 1000. Income is before-tax household income per year.
