# Supplementary material for: Socioeconomic inequalities, psychosocial stressors at work and physician-diagnosed depression: Time-to-event mediation analysis in the presence of time-varying confounders
Source: PLoS One. 2023 Oct 25;18(10):e0293388. doi: 10.1371/journal.pone.0293388 (PMC10599565; doi:10.1371/journal.pone.0293388)
Supplement: S8 Table — All values are HR, adjusted for age and sex. Bold: 95% CI that do not include 1. TSD: CAD 1000. Income is before-tax household income per year. (PDF) [file pone.0293388.s010.pdf]

S8 Table. Association between SES and psychosocial stressors at work in both men and women (n = 5898 complete cases).

SES	Job strain T_2	Job strain T_2 adjusted for job strain in T_1	ERI T_2	ERI T_2 adjusted for job strain in T_1
**Education**				
Ref: university	1	1	1	
2 years college	1.123 (0.966-1.342)	1.083 (0.935-1.227)	**0.849** (0.732-0.960)	**0.827** (0.733-0.928)
no college	1.084 (0.901-1.226)	1.094 (0.915-1.294)	**0.613** (0.524-0.737)	**0.613** (0.524-0.737)
**Income**				
Ref: ≥70 TSD	1	1	1	
40-70 TSD	**1.193** (1.035-1.383)	1.203 (0.981-1.409)	**0.825** (0.714 0.957)	**0.824** (0.729-0.952)
< 40 TSD	**1.487** (1.279 1.734)	**1.478** (1.176-1.850)	0.996 (0.836-1.188)	0.985 (0.819-1.165)
**Occupation**				
Ref: managers	1	1	1	1
professionals	**1.531** (1.169-2.154)	**1.513** (1.063-2.124)	**0.744** (0.623-0.907)	**0.729** (0.581-0.892)
others	**1.829** (1.408-2.627)	**1.820** (1.251-2.472)	**0.722** (0.600-0.885)	**0.707** (0.558-0.865)

All values are HR, adjusted for age and sex. Bold: 95% CI that do not include 1. TSD: CAD 1000. Income is before-tax household income per year.
