# Supplementary material for: Socioeconomic inequalities, psychosocial stressors at work and physician-diagnosed depression: Time-to-event mediation analysis in the presence of time-varying confounders
Source: PLoS One. 2023 Oct 25;18(10):e0293388. doi: 10.1371/journal.pone.0293388 (PMC10599565; doi:10.1371/journal.pone.0293388)
Supplement: S11 Table — All values are HR, adjusted for age, sex, job strain at T1, family indicators (marital status, presence of children in the household) and lifestyle habits (smoking, alcohol consumption and leisure time physical activity) at T1 and T2. Bold: 95% CI that do not include 1. TSD: CAD 1000. Income is before-tax household income per year. IE: Interventional effect. (PDF) [file pone.0293388.s013.pdf]

S11 Table. Contribution of psychosocial stressors at work to the socioeconomic gradient of depression in both men and women (n = 5898 complete cases).

SES	Job strain				ERI			
	**Total IE**	**Direct IE**	**Indirect IE**	**%**	**Total IE**	**Direct IE**	**Indirect IE**	**%**
**Education**								
Ref: univ.	1				1			
2 yr college	0.921	0.912	1.009	-	0.942	0.950	0.992	-
	(0.635-1.409)	(0.633-1.385)	(0.996-1.029)		(0.664-1.350)	(0.672-1.354)	(0.967 1.013)	
no college	1.147	1.142	1.004	2.6	1.086	1.134	0.958	-
	(0.783-1.680)	(0.780-1.676)	(0.995-1.020)		(0.787-1.524)	(0.822 1.603)	(0.921 0.986)	
**Income**								
Ref:≥70TSD	1				1			
40-70 TSD	1.037	1.027	1.009	27	0.933	0.954	0.977	-
	(0.703-1.611)	(0.699-1.590)	(0.996-1.033)		(0.654-1.374)	(0.665 1.387)	(0.947-0.998)	
< 40 TSD	1.424	1.406	1.012	4.2	1.297	1.298	0.999	-
	(0.959-2.227)	(0.951-2.194)	(0.993-1.040)		(0.902-1.891)	(0.906 1.905)	(0.974-1.025)	
**Occupation**								
Ref: manag.	1				1			
profession.	0.871	0.858	1.014	-	1.043	1.085	0.962	-
	(0.381-3.637)	(0.373-3.603)	(0.986-1.066)		(0.540-2.918)	(0.559-2.992)	(0.898-1.008)	
others	0.959	0.935	1.025	-	1.131	1.185	0.955	-
	(0.445-3.950)	(0.418-3.939)	(0.984-1.092)		(0.610-3.241)	(0.640 3.295)	(0.888-1.000)	
**Comb. SES**								
Ref: high	1				1			
median	1.580	1.574	1.003	0.9	1.206	1.215	0.993	-
	(0.941-2.972)	(0.942-2.984)	(0.983-1.028)		(0.786-2.281)	(0.790-2.270)	(0.960-1.023)	
low	**1.781**	**1.776**	1.003	0.7	1.381	1.436	**0.962**	-
	(1.086-3.286)	(1.080-3.275)	(0.984-1.025)		(0.901-2.557)	(0.943-2.619)	(0.921-0.992)	

All values are HR, adjusted for age, sex, job strain at T1, family indicators (marital status, presence of children in the household) and lifestyle habits (smoking, alcohol consumption and leisure time physical activity) at T1 and T2. Bold: 95% CI that do not include 1. TSD: CAD 1000. Income is before-tax household income per year. IE: interventional effect.
