# Supplementary material for: Socioeconomic inequalities, psychosocial stressors at work and physician-diagnosed depression: Time-to-event mediation analysis in the presence of time-varying confounders
Source: PLoS One. 2023 Oct 25;18(10):e0293388. doi: 10.1371/journal.pone.0293388 (PMC10599565; doi:10.1371/journal.pone.0293388)
Supplement: S13 Table — All values are HR, adjusted for age, job strain at T1, family indicators (marital status, presence of children in the household) and lifestyle habits (smoking, alcohol consumption and leisure time physical activity) at T1 and T2. Bold: 95% CI that do not include 1. TSD: CAD 1000. Income is before-tax household income per year. IE: Interventional effect. (PDF) [file pone.0293388.s015.pdf]

S13 Table. Total and direct effect estimates of socioeconomic status on depression and indirect effect estimates mediated through psychosocial stressors at work in women after excluding prevalent cases since 1991.

SES	Job strain				ERI			
	**Total IE**	**Direct IE**	**Indirect IE**	**%**	**Total IE**	**Direct IE**	**Indirect IE**	**%**
**Education**								
Ref: univ.	1				1			
2 yr college	0.710 (0.455-1.105)	0.704 (0.450-1.099)	1.009 (0.990-1.027)	-	0.750 (0.511-1.100)	0.761 (0.519-1.116)	0.985 (0.958-1.014)	-
no college	0.833 (0.544-1.277)	0.830 (0.542-1.273)	1.004 (0.992-1.016)	-	0.814 (0.551-1.201)	0.857 (0.580-1.266)	0.950 (0.910-0.991)	-
**Income**								
Ref:≥70TSD	1				1			
40-70 TSD	0.883 (0.555-1.404)	0.877 (0.550-1.398)	1.007 (0.990-1.024)	-	0.818 (0.533-1.254)	0.843 (0.552-1.286)	0.971 (0.939-1.003)	-
< 40 TSD	1.093 (0.670-1.781)	1.082 (0.662-1.770)	1.009 (0.988-1.031)	11	1.011 (0.665-1.539)	1.015 (0.668-1.541)	0.997 (0.965-1.029)	-
**Occupation**								
Ref: manag.	1				1			
profession.	1.009 (0.228-4.468)	0.991 (0.221-4.448)	1.019 (0.954-1.088)	-	1.603 (0.419-6.124)	1.656 (0.436-6.297)	0.967 (0.906-1.033)	-
Others	0.810 (0.191-3.435)	0.774 (0.176-3.407)	1.047 (0.961-1.140)	-	1.337 (0.358-4.991)	1.394 (0.377-5.163)	0.959 (0.895-1.027)	-

All values are HR, adjusted for age, job strain at T1, family indicators (marital status, presence of children in the household) and lifestyle habits (smoking, alcohol consumption and leisure time physical activity) at T1 and T2. Bold: 95% CI that do not include 1. TSD: CAD 1000. Income is before-tax household income per year. IE: interventional effect.
