# Supplementary material for: Socioeconomic inequalities, psychosocial stressors at work and physician-diagnosed depression: Time-to-event mediation analysis in the presence of time-varying confounders
Source: PLoS One. 2023 Oct 25;18(10):e0293388. doi: 10.1371/journal.pone.0293388 (PMC10599565; doi:10.1371/journal.pone.0293388)
Supplement: S1 Appendix — (PDF) [file pone.0293388.s018.pdf]

Mathematical Appendix for manuscript "Socioeconomic inequalities, psychosocial stressors at work and physician-diagnosed depression: a time-to-event mediation analysis."

Ana Paula Bruno Pena-Gralle, Denis Talbot, Xavier Trudel, Alain Milot, Mahée Gilbert-Ouimet, Mathilde Lavigne-Robichaud, Ruth Ndjaboué, Alain Lesage, Sophie Lauzier, Michel Vézina, Johannes Siegrist and Chantal Brisson

In this Appendix, we present the mathematical development of the statistical approach that was used to perform our mediation analysis. This approach is an adaptation for time-to-event outcomes of the method proposed in VanderWeele and Tchetgen Tchetgen (2017) Mediation analysis with time varying exposures and mediators. Journal of the Royal Statistical Society : Series B. 79; 917-938.

We f rst introduce some notation to describe the data structure. Let L_0 and L_1 be pre-exposure and pre-mediator covariates, A the exposure, M the mediator and Y the time-to-event. We assume that independant and identically distributed data $i = 1, \ldots, n$ are sampled from a given population. The directed acyclic graph in Figure 1 represents the causal structure we assume.

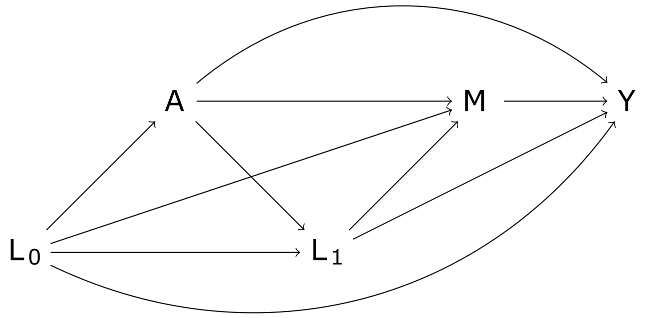

FIGURE 1 – Directed acyclic graph depicting the assumed causal structure

We now introduce further notation in order to def ne our causal parameter of interest. Let G be the distribution of the mediator. We denote by M_a the counterfactual value that the mediator would have had if the exposure had taken value $A = a$ perhaps contrary to the fact, G_a the distribution of M_a, and $\lambda_{am}(t)$ the counterfactual outcome hazard at time t that would have been observed had the exposure taken value $A = a$ and the mediator taken value $M = m$. We def ne $\lambda_{aG_{a^0}}(t)$, the counterfactual hazard at time t had the exposure taken value $A = a$ and the mediator value was randomized according to the distribution G_{a^0}. The causal parameters of interest are the interventional direct effect (IDE) and the interventional indirect effect (IIE) comparing exposure level $A = a$ with a level of reference $A = a^0$:

— IDE(t) = $\frac{\lambda_{aG_{a'}}(t)}{\lambda_{a'G_{a'}}(t)}$

— IIE(t) = $\frac{\lambda_{aG_a}(t)}{\lambda_{aG_{a'}}(t)}$.

The IDE represents the outcome hazard ratio under a scenario where exposure takes level $A = a$ and the mediator is randomized to $G_{a'}$ versus a scenario where exposure takes level a' and the mediator is again randomized to $G_{a'}$. As such, the IDE isolates the direct effect of the exposure $A = a$ versus $A = a'$, since the mediator is assigned the same distribution under both scenarios. The IIE represents the outcome hazard ratio under a scenario where exposure takes level $A = a$ and mediator is randomized to G_a versus a scenario where the exposure takes level $A = a'$ and mediator is randomized to G'_a. The IIE thus estimates the effect of the exposure on the outcome that is attributable to its impact on the mediator (i.e., its indirect effect), since both scenarios only differ in their distribution of the mediator.

To estimate these causal quantitites, we consider a marginal structural Cox model for the outcome

$$\lambda_{am}(t) = \lambda_0(t) \exp(\theta_1 a + \theta_2 m)$$

and a marginal structural model for the mediator

$$h[\mathbb{E}(M_a)] = \beta_0 + \beta_1 a,$$

where h is a link function. If M is a binary variable, h could be the logit function, for example. Note that the outcome model could feature an interaction term between the exposure and the outcome. As will be seen below, the IDE and IIE can be expressed as a function of the parameters of these marginal structural models. The parameters of these marginal structural models can themselves be estimated using various estimators, such as g-computation, inverse probability weighting or targeted maximum likelihood. In this manuscript, we have used an inverse probability weighting estimator. We consider the following weights

$$w_i^A = \frac{1}{P(A = a_i | L_{0i})}$$
$$w_i^M = \frac{1}{P(M = m_i | L_{1i}, A_i, L_{0i})}.$$

Under the usual causal assumptions of exchangeability (no unmeasured confounders of either the exposure-mediator, exposure-outcome or mediator-outcome relations), positivity and consistency, the parameters of the outcome marginal structural model can be consistently estimated by fitting the weighted Cox regression $\lambda(t|A, M) = \lambda_0(t) \exp(\theta A + \theta M)$ weighting observations according to the weights $w_i^A \times w_i^M$. Estimates of the baseline hazard $\lambda_0(t)$ are also required, which is readily available

in most statistical software performing Cox regressions. The parameters of the mediator marginal structural models can be consistently estimated by fitting the weighted generalized linear model $h[\mathbb{E}(M|A)] = \beta_0 + \beta_1 A$ with weights w_i^A.

We now show that the IDE and IIE can expressed as a function of the parameters of the preceding marginal structural models.

$$
\begin{aligned}
\lambda_{aG_{a'}}(t) &= \frac{f_T(t|A=a, M=G_{a'})}{S_T(t|A=a, M=G_{a'})} \\
f_T(t|A=a, M=G_{a'}) &= \int_m f_T(t|A=a, M=m) dF_{M_{a'}}(m) \\
&= \int_m \lambda_0(t) \exp(\theta_1 a + \theta_2 m) \exp\left(-\Lambda_0(t) \exp\left[\theta_1 a + \theta_2 m\right]\right) dF_{M_{a'}}(m) \\
S_T(t|A=a, M=G_{a'}) &= \int_m \exp\left(-\Lambda_0(t) \exp\left[\theta_1 a + \theta_2 m\right]\right) dF_{M_{a'}}(m) \\
\frac{f_T(t|A=a, M=G_{a'})}{S_T(t|A=a, M=G_{a'})} &= \frac{\int_m \lambda_0(t) \exp(\theta_1 a + \theta_2 m) \exp\left(-\Lambda_0(t) \exp\left[\theta_1 a + \theta_2 m\right]\right) dF_{M_{a'}}(m)}{\int_m \exp\left(-\Lambda_0(t) \exp\left[\theta_1 a + \theta_2 m\right]\right) dF_{M_{a'}}(m)} \\
&= \frac{\lambda_0(t) \exp(\theta_1 a) \exp\left(-\Lambda_0(t) \exp[\theta_1 a]\right) \int_m \exp(\theta_2 m) \exp\left(-\Lambda_0(t) \exp\left[\theta_2 m\right]\right) dF_{M_{a'}}(m)}{\exp(-\Lambda_0(t) \exp[\theta_1 a]) \int_m \exp\left(-\Lambda_0(t) \exp\left[\theta_2 m\right]\right) dF_{M_{a'}}(m)} \\
&= \frac{\lambda_0(t) \exp(\theta_1 a) \int_m \exp(\theta_2 m) \exp\left(-\Lambda_0(t) \exp\left[\theta_2 m\right]\right) dF_{M_{a'}}(m)}{\int_m \exp\left(-\Lambda_0(t) \exp\left[\theta_2 m\right]\right) dF_{M_{a'}}(m)},
\end{aligned}
$$

where $\Lambda_0(t) = \int_0^t \lambda_0(t) dt$ is the cumulative hazard function, and $dF_{M_a}(m)$ is an appropriate measure relative to M. For example, if M is a categorical variable with possible values $m_0, ..., m_k$, then the integral becomes $\sum_{m=m_0,...,m_k}$ and $dF_{M_a}(m) = P(M_a = m)$. If M is continuous, then $dF_{M_a}(m) = g_a(m) dm$, where $g_a(m)$ is the density of M_a at m.

As a result of the previous developments, the IDE and IIE can be expressed as

$$
\begin{aligned}
IDE(t) &= \frac{\dfrac{\lambda_0(t) \exp(\theta_1) \int_m \exp(\theta_2 m) \exp(-\Lambda_0(t) \exp[\theta_2 m]) dF_{M_0}(m)}{\int_m \exp(-\Lambda_0(t) \exp[\theta_2 m]) dF_{M_0}(m)}}{\dfrac{\lambda_0(t) \int_m \exp(\theta_2 m) \exp(-\Lambda_0(t) \exp[\theta_2 m]) dF_{M_0}(m)}{\int_m \exp(-\Lambda_0(t) \exp[\theta_2 m]) dF_{M_0}(m)}} \\
&= \exp(\theta_1),
\end{aligned}
$$

and

$$
\begin{aligned}
IIE(t) &= \frac{\dfrac{\lambda_0(t) \exp(\theta_1) \int_m \exp(\theta_2 m) \exp(-\Lambda_0(t) \exp[\theta_2 m]) dF_{M_1}(m)}{\int_m \exp(-\Lambda_0(t) \exp[\theta_2 m]) dF_{M_1}(m)}}{\dfrac{\lambda_0(t) \exp(\theta_1) \int_m \exp(\theta_2 m) \exp(-\Lambda_0(t) \exp[\theta_2 m]) dF_{M_0}(m)}{\int_m \exp(-\Lambda_0(t) \exp[\theta_2 m]) dF_{M_0}(m)}} \\
&= \frac{\dfrac{\int_m \exp(\theta_2 m) \exp(-\Lambda_0(t) \exp[\theta_2 m]) dF_{M_1}(m)}{\int_m \exp(-\Lambda_0(t) \exp[\theta_2 m]) dF_{M_1}(m)}}{\dfrac{\int_m \exp(\theta_2 m) \exp(-\Lambda_0(t) \exp[\theta_2 m]) dF_{M_0}(m)}{\int_m \exp(-\Lambda_0(t) \exp[\theta_2 m]) dF_{M_0}(m)}}.
\end{aligned}
$$

Note that if different structural models were assumed, for example by including a $a \times m$ term in the outcome model, the final expressions would be slightly different.
